# High-throughput drug screening to investigate blood-brain barrier permeability *in vitro* with a focus on breast cancer chemotherapeutic agents

**DOI:** 10.3389/fddev.2024.1331126

**Published:** 2024-06-27

**Authors:** Carolin J. Curtaz, Sophia Wucherpfennig, Emad Al-Masnaea, Saskia-Laureen Herbert, Achim Wöckel, Patrick Meybohm, Malgorzata Burek

**Affiliations:** ^1^ University Hospital Würzburg, Department of Gynecology and Obstetrics, Würzburg, Germany; ^2^ University Hospital Würzburg, Department of Anaesthesiology, Intensive Care, Emergency and Pain Medicine, Würzburg, Germany

**Keywords:** metastatic breast cancer, blood-brain barrier, *in vitro* models, high-throughput drug screening, GW2974, 4-amino-1,8-naphthalimide

## Abstract

Therapy of cerebral diseases such as brain metastatic breast cancer is still challenging. Due to the blood-brain barrier (BBB), a tight barrier that protects the brain and prevents the passage of many drugs, therapeutically sufficient drug concentrations in the brain are often not achieved. Therefore, methods and drugs to manipulate the BBB permeability are required. Here we used high-throughput screening (HTS) to identify chemicals that may increase BBB permeability. Human BBB *in vitro* model derived from hematopoietic CD34^+^ stem cells (differentiated to brain-like endothelial cells, BLECs) was used. BLECs were seeded on 96-well plates coated with biotinylated gelatin, treated with respective chemicals for 24 h followed by addition of FITC-avidin for permeability estimation. Selected substances were further tested *in vitro* on BLECs. Cell viability, gene and protein expression were measured using CellTiter-Glo^®^, qPCR and Western blot, respectively. From 1,278 compounds, we identified 175 substances that cause at least a 50 percent increase in BBB permeability. Two substances from the substance classes used in breast cancer therapy, GW2974 (tyrosine kinase inhibitor) and 4-amino-1,8-naphthalimide (ANI) (PARP inhibitor), were analyzed in more detail. ANI was nontoxic to BLECs, while GW2974 decreased or increased viability depending on the concentration used. Both compounds significantly increased BBB permeability and altered protein and mRNA expression in BLECs. Influencing the BBB permeability in patients with brain metastases could increase the response rate to systemic therapy. Using HTS, we were able to accurately and quickly identify compounds that increase BBB permeability and show that using this type of screening method can be applied to endothelial paracellular permeability testing.

## 1 Introduction

Brain metastases are the most common cause of malignant tumors of the central nervous system (CNS) - even four times more common than primary brain tumors. Breast cancer is the most common cancer in women in the western world and the second most common solid tumor with brain metastases after lung, followed by melanoma (Erdmann et al., 2021). Its incidence has increased in recent years. This is related to improved technical possibilities of metastasis diagnosis such as contrast-enhanced magnetic resonance imaging, but also to the improving treatment options, especially systemic therapy. 15%–30% of breast cancer patients develop brain metastases during the course of their disease (Rick et al., 2019). Clinically, brain metastases from breast cancer are associated with a significant reduction in live expectancy and quality of live. The reason for this is that, apart from pronounced neurological symptoms and the current therapeutic methods consisting of metastasis removal and whole-brain irradiation, there are no outstanding systemic therapeutic agents that inhibit the intracerebral growth of brain metastases (Witzel et al., 2016; Meattini et al., 2020).

The blood-brain barrier (BBB), composed of the brain microvascular endothelial cells, pericytes and astrocytes, prevents many drugs from entering the brain. The endothelial cells form a tight and selective barrier through tight (TJs) and adherens junctions (AJs), limited transcytosis and expression of specific transporter molecules (Abbott et al., 2010; Kadry et al., 2020; Schick et al., 2021; Sun et al., 2022). Because the BBB is impermeable to most systemically applied therapeutics, therapeutic drug concentrations in the brain are often too low without manipulation of the barrier (Krizbai et al., 2016; Watson et al., 2023).

For this reason, researchers are still searching for methods and drugs to affect the permeability of the BBB and the effectiveness of CNS drug delivery. Specific chemicals or substances affecting TJs, solute carrier transportrs and active efflux pumps could therefore offer possible starting points (Puris et al., 2022; Puris et al., 2023).

Brain tumors and tumor metastases form the so-called blood-tumor barrier, which is usually more permeable than the normal BBB. However, studies in patients with gliomas have shown that the barrier remained intact at the invasive margins of the gliomas (Gao et al., 2018; Pacheco et al., 2022). Even with micrometastases (<1 mm), the BBB is normal and therefore protective against most anticancer drugs (Soffietti et al., 2020). In addition, it shows great heterogeneity in permeability from lesion to lesion and from region to region of the same lesion (Lockman et al., 2010).

Therefore, the permeability of the blood-tumor barrier must also be increased in order to ensure the intracerebral use of systemic therapy. It is desirable to cause a targeted change in permeability only in the tumor area. For example, it could be shown in a mouse model that ATP-sensitive potassium channels are overexpressed in the blood-tumor barrier, while they are hardly detectable in normal brain endothelial cells. If these are treated with, e.g., minoxidil sulfate, the transport of antineoplastic drugs such as carboplatin or HER2 antibodies directly into the tumor area is facilitated (Ningaraj et al., 2003).

In general, high-throughput drug screening (HTS) is used to discover and develop new drugs. Many different test substances are examined within a short period of time using defined assays (Hertzberg and Pope, 2000).

Here we therefore establish and validate a suitable experimental setup for a HTS to screen a variety of compounds for their effect on BBB permeability *in vitro*. In preliminary work, Salvador et al. established a permeability assay without the need to use transwell inserts, which was modified here (Salvador et al., 2015). We used human CD34^+^ hematopoietic stem cells differentiated into brain like endothelial cells (BLECs) in 96-well plates to identify those agents that affect the paracellular permeability of the BBB and could therefore potentially be used in clinical practice to increase brain penetration of systemically administered drugs.

## 2 Materials and methods

### 2.1 Cell culture

CD34^+^ hematopoietic stem cells were isolated from umbilical cord blood and expanded in Endothelial Cell Basal Medium supplemented with Microvascular Endothelial Cell Growth Supplement Kit (PLEOBiotechm, PB-BH-100-9806, PB-SH-100–4099) on gelatin-coated plates as described previously (Cecchelli et al., 2014; Curtaz et al., 2020). Cells from passage 6 were differentiated into BLECs in co-culture with brain pericytes as previously described (Curtaz et al., 2020; Nishihara et al., 2020). Briefly, pericytes were cultured in DMEM low glucose (Sigma-Aldrich, D6046) supplemented with 20% FCS, 2% L-glutamine and 1% penicillin-streptomycin. For differentiation, CD34^+^ hematopoietic stem cells were plated in Matrigel-coated transwells (pore-size 0.4 µm, Corning) and co-cultured with pericytes in Endothelial Cell Basal Medium with supplements for 5 days. On 96-well plates, CD34^+^ hematopoietic stem cells were cultured in pericytes-conditioned Endothelial Cell Basal Medium for 5 days.

### 2.2 High-throughput drug screening (HTS)

We used the Library of Pharmacologically Active Compounds (Sigma-Aldrich, LO1280) to screen their effects on BBB permeability *in vitro*. The compound library was stored at −20°C. Compounds dissolved in DMSO at a concentration of 10 mM were diluted to 100 µM with cell culture medium. The 80 diluted compounds from each rack were pipetted onto the experimental plate according to their rack position, 100 µL per well. Each drug rack was tested on two experimental plates. Two compounds, 4-amino-1,8-naphthalimide (ANI, Sigma-Aldrich, A0966) and GW2974 (Sigma-Aldrich, G0668) were selected for more detailed analysis. Stock solutions of ANI and GW2974 were prepared at 10 mM in DMSO.

### 2.3 Measurement of endothelial permeability on 96-well plates

Endothelial permeability on 96-well plates was measured as previously described with some modifications (Salvador et al., 2015). Briefly, 96-well plates were coated with 100 µL of 0.25 mg/mL biotin conjugated-gelatin overnight and then washed twice with PBS. Endothelial cells (3 × 10^4^) were plated in pericyte-conditioned medium and the cells were grown to confluence for 6 days followed by the treatment with compounds for 24 h. On day seven, 6 μg/mL of NeutrAvidin ™, FITC conjugate (FITC-avidin, 60 kDa) (Thermo Fisher Scientific) was added to cells for 3 min. The cells were washed twice with PBS to remove the unbound FITC-avidin. Wells were filled with 100 µL of PBS and bound FITC-avidin fluorescence was measured using plate reader with wavelengths of 492 nm. The fluorescence units of cells treated with compounds were normalized to values of cells treated with vehicle (DMSO) and presented as average with standard deviation.

### 2.4 Cell viability assay

CellTiter-Glo 2.0 Viability Assay (Promega, G9241) was used to estimate the cell viability. 3 × 10^4^ cells per well were plated in gelatin-coated 96-well plates as described above. The cells were treated with chemicals for 24 h. Cell viability was measured according to the manufacturer’s instruction. Eight technical replicates were used per treatment. The experiments were repeated three times.

### 2.5 Transendothelial electrical resistance and paracellular permeability measurement in transwell

To validate the results obtained in 96-well plates, we used transwells (12 well, pore size 0.4 µm, Corning) and smaller size tracer, fluorescein (376 Da) for selected compounds. Transendothelial electrical resistance (TEER) and paracellular permeability to fluorescein were measured as described previously (Curtaz et al., 2020; Salvador et al., 2021; Li et al., 2023). Briefly, transwells containing confluent and differentiated BLECs were initially used for TEER measurement. The TEER was measured with a chopstick electrode (Millipore). The values of the blank filters were subtracted from the values of the transwells with cells and multiplied by the transwell surface area (1.12 cm^2^). The transwells were then placed in a 12-well plate containing 1.5 mL HEPES-buffered Ringer’s solution (pH 7.4) (lower compartment). Five hundred µl of 1 mM fluorescein solution (Sigma-Aldrich) was added to the transwell (upper compartment). Every 20 min during the 1-h test, inserts were placed in a new well with fresh buffer solution and aliquots from the lower and the upper compartment were measured at 490 nm with a Tecan Microplate Reader (Thermo Fisher Scientific). For each condition, three inserts with and without cells were measured and used to calculate the permeability coefficient (Pe). The experiments were repeated three times.

### 2.6 Western blot analysis

Western blot analysis was performed as recently described (Dilling et al., 2017; Kaiser et al., 2018; Burek et al., 2020; Reschke et al., 2022). The cells were cultured as described above and treated with chemicals in pericyte-conditioned medium. Control cells were treated with DMSO for 24 h followed by protein extraction. Protein levels were determined using the BCA Protein Assay Kit (Thermo Fisher Pierce) and 30 µg of protein were loaded on a gel. After the transfer of proteins to PVDF membrane (Bio-Rad Laboratories), the membranes were incubated with the respective primary antibody diluted in PBS containing 1% Bovine Serum Albumin (Sigma-Aldrich) at 4°C overnight. Following antibodies were used: mouse-anti-β-actin-HRP conjugated (1:20000, Sigma- Aldrich, A3854), mouse-anti-BCRP monoclonal antibody (1:100, Santa Cruz Biotechnology, SC-58222), rabbit-anti-Cldn5 polyclonal antibody (1:500, Thermo Fisher Scientific, 34–1,600), rabbit-anti-Glut-1 polyclonal antibody (1:2000, Sigma- Aldrich, 07–1,401), mouse anti-occludin monoclonal antibody (1:1,000, Thermo Fisher Scientific, 33–1,500), mouse anti-P-glycoprotein monoclonal antibody (1:50, Enzo, ALX-801-002), goat-anti-VE-cadherin polyclonal antibody (1:200, Santa Cruz Biontechnology, SC-6458), mouse-anti-ZO-1 monoclonal antibody (1:500, Thermo Fisher Scientific, 33–9,100). Respective secondary antibodies were used: horse anti-mouse IgG (1:3000, Cell Signaling Technology, 7076S), mouse anti-goat IgG (Santa Cruz Biotechnology, A1921), goat anti-rabbit IgG (1:3000, Cell Signaling Technology, 7074S). Images were acquired using the Enhanced Chemiluminescence Solution and the FluorChem FC2 Multi-Imager II (Alpha Innotech). ImageJ 1.50i software (National Institutes of Health) was used to quantify the band intensity.

### 2.7 Real-time qPCR

Real-time quantitative polymerase chain reaction (qPCR) was performed as previously described (Gerhartl et al., 2020; Curtaz et al., 2022; Feldheim et al., 2022). The NucleoSpin^®^ RNA kit (Macherey-Nagel, 740955.250) was used for the isolation of RNA according to manufacturer instructions. 1 μg of RNA was used for cDNA synthesis with High- Capacity cDNA Reverse Transcription Kit (Thermo Fisher Scientific, 4368813). TaqMan^®^ Fast Advanced Master Mix (Thermo Fisher Scientific, 4444965) and TaqMan^®^ Gene Expression Assays (Thermo Fisher Scientifc) were used in qPCR with QuantStudio 7 Flex System according to manufacture’s instructions. Following TaqMan^®^ Gene Expression Assays were used: ABCB1 (Hs00184500_m1), ABCG2 (Hs01053790_m1), CDH5 (Hs00901465_m1), CLDN5 (Hs00533949_m1), OCLN (Hs00170162_m1), SLC2A1 (Hs00892681_m1), TJP1 (Hs01551861_m1). CANX (Hs01558409_m1) was used as endogenous control. Three technical replicates were used per gene. The experiments were repeated three times. QuantStudio™ Real-Time PCR Software v1.7.1 (Thermo Fisher Scientific) was used for analysis.

### 2.8 Statistical analysis

The statistical analysis was carried out with GraphPad Prism 9. Data are presented as means from three independent experiments with standard deviations unless otherwise stated. ANOVA followed by Dunnett’s multiple comparisons test was used to determine statistical significance. Values were considered statistically significant when *p* < 0.05.

## 3 Results

### 3.1 High-throughput screening of pharmacologically active compounds and their effects on endothelial permeability

To quickly filter out compounds affecting endothelial cell permeability from all 1,278 compounds in the Library of Pharmacologically Active Compounds, a permeability test was performed in 96-well plates. To determine the test conditions, different FITC-avidin concentration were tested (results not shown). Optimal conditions were found for 6 μg/mL FITC-avidin. We found 175 compounds that cause at least a 0.5-fold increase in BBB permeability. Selected compounds that increase the permeability of BLECs are listed in Table 1. We selected two compounds, GW2974 (tyrosine kinase inhibitor) and 4-amino-1,8-naphthalimide (ANI) (PARP inhibitor), for further analysis because they belong to the class of clinically used compounds.

**TABLE 1:** List of compound that increase BBB permeability *in vitro*. Listed are selected substances from the Library of Pharmacologically Active Compounds that increase the permeability of brain like endothelial cells (BLECs) by at least 0.5-fold. All compounds were used at a concentration of 100 µM. DMSO was used as a control. The average x-fold fluorescence ±standard deviation normalized to the control is given.

-Fold FITC-avidin fluorescence	Name	Class
3.6 ± 0.49	SB 216763	Phosphorylation
3.5 ± 1.83	GW2974	Phosphorylation
3.4 ± 1.15	Idarubicin	DNA Metabolism
2.8 ± 0.55	4-Amino-1,8-naphthalimide	Apoptosis
2.1 ± 0.17	AGK2	Gene Regulation
2.1 ± 0.20	Stattic	Gene Regulation
2.0 ± 0.29	CP466722	Kinase/Phosphatase
2.0 ± 0.08	Rottlerin	Phosphorylation
1.9 ± 0.25	EMPA	Orexin
1.8 ± 0.20	Capsazepine	Vanilloid
1.8 ± 0.24	TH-257	Phosphorylation
1.7 ± 0.25	CGS-12066A maleate	Serotonergics
1.7 ± 0.21	Benztropine mesylate	Cell Signaling and Neuroscience
1.7 ± 0.22	Chlorcyclizine	Cell Signaling
1.7 ± 0.23	Brazilin	Gene Regulation
1.7 ± 0.29	SBI-0087702	Cell Signaling and Neuroscience
1.7 ± 0.33	Macelignan	Nitric Oxide
1.7 ± 0.22	Carvedilol	Adrenoceptor
1.6 ± 0.24	Diacylglycerol Kinase Inhibitor II	Phosphorylation
1.6 ± 0.29	Ebastine	Histaminergics
1.6 ± 0.13	KINK-1 hydrochloride	Phosphorylation
1.6 ± 0.27	R (+)-Butylindazone	Ion Pump
1.6 ± 0.20	Flupirtine maleate	Glutamatergics
1.6 ± 0.27	8-(3-Chlorostyryl)caffeine	Adenosine
1.5 ± 0.33	Flibanserin	Serotonergics
1.5 ± 0.31	Azoramide	Cell Signaling
1.5 ± 0.23	ML396	Neurotransmission
1.5 ± 0.01	Reserpine	Serotonergics
1.5 ± 0.17	Lomeguatrib	Gene Regulation
1.5 ± 0.24	ET-18-OCH3	Lipid

### 3.2 Concentration-response analysis of ANI and GW2974 on endothelial viability and barrier properties

**FIGURE 1:**
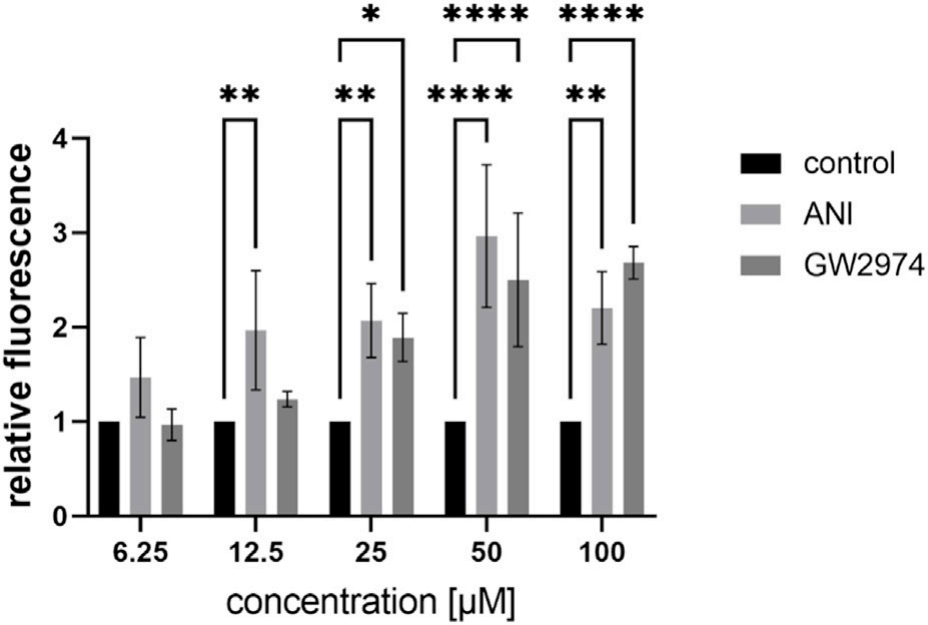
Effects of low concentrations of 4-amino-1,8-naphthalimide (ANI) and GW2974 on the FITC-avidin permeability of brain like endothelial cells (BLECs). Differentiated BLECs were treated with 6.25 µM, 12.5 µM, 25 µM, 50 µM and 100 µM of ANI or GW2974 for 24 h in 96-well plates. The fluorescence of FITC-avidin bound to biotinylated-gelatine was measured and is shown as fold of the cells treated with the respective DMSO concentration, *n* = 3, *p < 0.05, **p < 0.01, ****p < 0.0001.

In the HTS, a concentration of 100 µM for all compounds was used. To test lower concentrations of ANI and GW2974, we further diluted the stock solution and treated the cells with 6.25 µM, 12.5 µM, 25 µM, 50 µM and 100 µM for 24 h and used the respective DMSO concentrations as a control (Figure 1). Treatment with 6.25 µM of ANI and GW2974 had no effect on endothelial permeability. ANI at concentrations from 12.5 µM to 100 µM significantly increased endothelial permeability (2.0-, 2.1-, 3- and 2.2-fold, respectively, *p* < 0.05). GW2974 increased endothelial permeability at concentration of 25, 50 and 100 µM (1.9-, 2.5- and 2.7-fold, respectively, *p* < 0.05) (Figure 1). These results suggest that the effects of ANI and GW2974 are concentration dependent, with higher concentrations showing greater effects on endothelial permeability.

**FIGURE 2:**
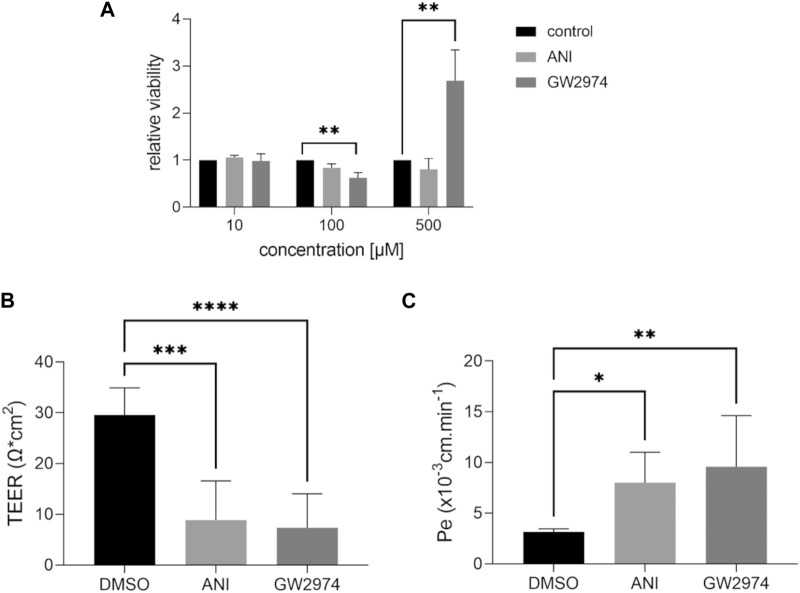
Effects of 4-amino-1,8-naphthalimide (ANI) and GW2974 on cell viability and barrier properties of brain like endothelial cells (BLECs). Cell viability was estimated after treatment with 10 µM, 100 µM and 500 µM ANI or GW2974 for 24 h **(A)**, *n* = 3. Transendothelial electrical resistance (TEER) **(B)** and paracellular permeability to fluorescein **(C)** were measured in transwells after treatment of cells with 100 µM ANI or 100 µM GW2974 for 24 h, *n* = 3, *p < 0.05, **p < 0.01, ***p < 0.001, ****p < 0.0001.

Cell viability assays were performed using CellTiter-Glo 2.0. BLECs were treated with 100 µM of ANI and GW2974 as used in HTS and lower (10 µM) and higher (500 µM) concentrations and corresponding DMSO controls (Figure 2A). ANI had no effect on endothelial cell viability at any concentration, while GW2974 resulted in a 0.6-fold decrease (*p* < 0.01) in cell viability at 100 µM and 2.7-fold (*p* < 0.01) increased cell viability/higher levels of ATP at 500 µM (Figure 2A).

### 3.3 ANI and GW2977 compromise barrier properties of BLECs

To validate the results obtained in 96-well system, measurements were carried out in the transwell insert with a small tracer fluorescein. TEER, an additional parameter of endothelial barrier integrity, can be measured in addition to paracellular permeability to fluorescein due to the two-chamber system when using transwell (Figure 2B,C). Treatment with 100 µM ANI and GW2974 resulted in a significant decrease in TEER (3.2- and 4-fold, *p* < 0.001) and a significant increase in paracellular permeability (2.6- and 3-fold, respectively, *p* < 0.05) consistent with the results obtained in HTS.

### 3.4 Influence of GW2974 and ANI on the expression of endothelial cell markers

**FIGURE 3:**
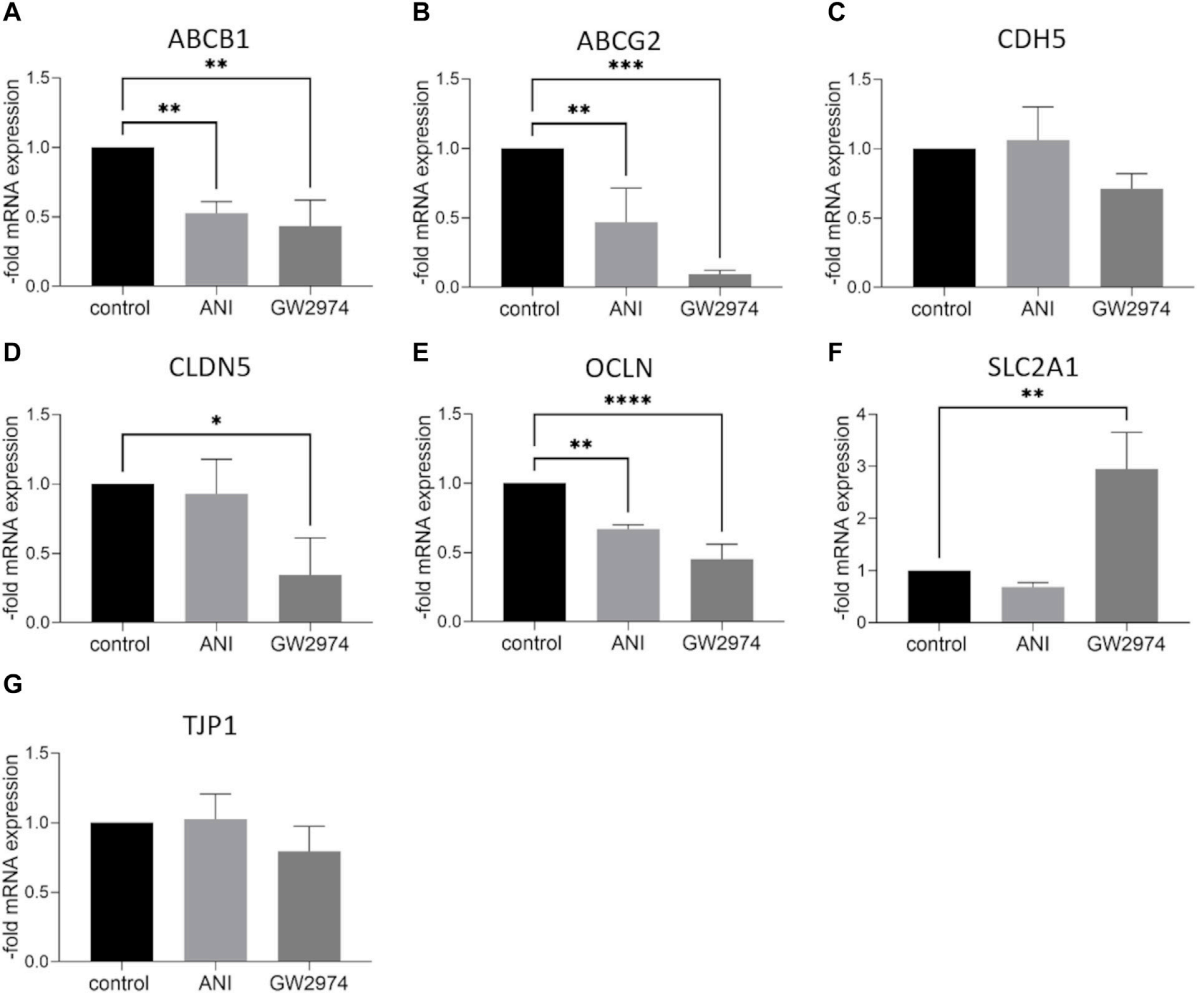
Effect of 4-amino-1,8-naphthalimide (ANI) and GW2974 on mRNA expression of endothelial markers. Brain like endothelial cells (BLECs) were treated with 100 µM ANI or 100 µM GW2974 for 24 h followed by qPCR for ATP Binding Cassette Subfamily B Member 1 (ABCB1) **(A)**, ATP Binding Cassette Subfamily G Member 2 (ABCG2) **(B)**, VE-cadherin (CDH5) **(C)**, claudin-5 (CLDN5) **(D)**, occludin (OCLN) **(E)**, Solute Carrier Family 2 Member 1 (SLC2A1) **(F)** and Tight Junction Protein 1 (TJP1) **(G)**. The means and the standard deviation (*n* = 3) are shown as the fold of the control cells. *p < 0.05, **p < 0.01, ***p < 0.001, ****p < 0.0001.

**FIGURE 4:**
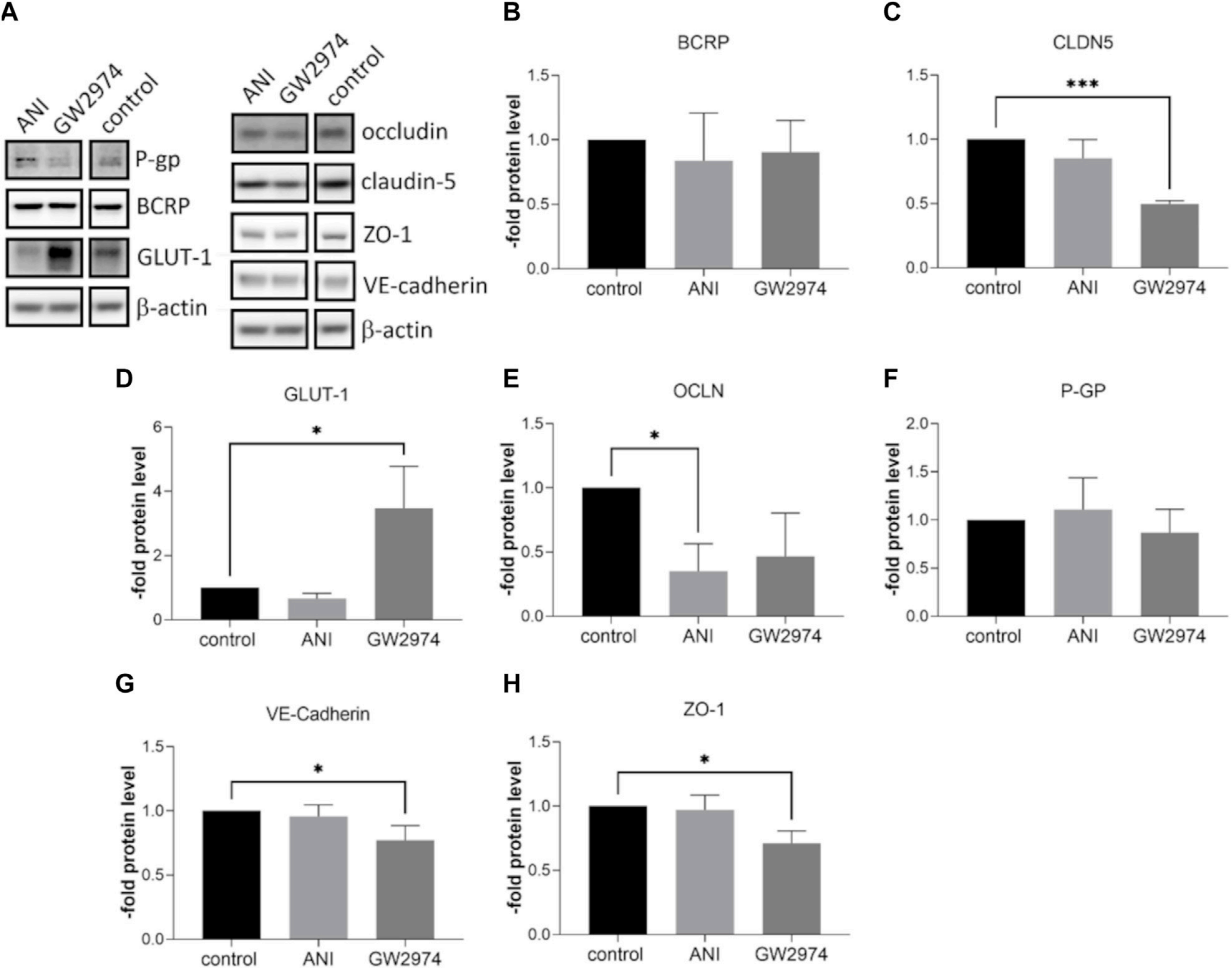
Effects of 4-amino-1,8-naphthalimide (ANI) and GW2974 on protein level of endothelial markers. Brain like endothelial cells (BLECs) were treated with 100 µM ANI or 100 µM GW2974 for 24 h followed by Western blot **(A)** and its densitometric analysis of Breast cancer resistance protein (BCRP) **(B)**, claudin-5 (CLDN5) **(C)**, glucose transporter-1 (GLUT-1) **(D)**, occludin (OCLN) €, P-glycoprotein (P-GP) **(F)**, VE-cadherin **(G)** and Zonula occludens-1 (ZO-1) **(H)**. The means and the standard deviation (n = 3) are shown as the fold of the control cells. *p < 0.05, ***p < 0.001.

At the mRNA level, the expression of ABC transporter, ABCB1 and ABCG2 was significantly lower in BLECs treated with 100 µM ANI (0.5-fold, *p* < 0.01) and GW2974 (0.4-fold, *p* < 0.05). A similar effect was observed for the tight junction protein occludin mRNA (0.7-fold and 0.45-fold, respectively, *p* < 0.01). CLDN5 mRNA was downregulated after treatment with GW2974 (0.3-fold, *p* < 0.05) while the mRNA expression of glucose transporter SLC2A1 was significantly upregulated after GW2974 treatment (2.95-fold, *p* < 0.01). No significant changes in CDH5 und TJP1 mRNA expression were observed (Figures 3A–G). At the protein level, claudin-5 was 0.5-fold (*p* < 0.001), VE-cadherin 0.7-fold (*p* < 0.05) and ZO-1 0.7-fold (*p* < 0.05) downregulated in cells treated with 100 µM GW2974, while treatment with 100 µM ANI only resulted in a reduction in occludin protein levels (0.35-fold, *p* < 0.05) (Figures 4A, C, E, G, H). GLUT-1 (SLC2A1) protein level was significantly increased in GW2974-treated cells (3.5-fold, *p* < 0.05), while no effect of ANI was observed (Figures 4A, D). Both treatments had no effect on the protein levels of the efflux pumps BCRP (ABCG2) and P-GP (ABCB1) (Figures 4A, B, F).

## 4 Discussion

In the treatment of neurological diseases, including breast cancer brain metastases, strategies to increase CNS drug delivery via the BBB are urgently needed. One possible approach to achieve this is to temporarily increase BBB permeability through the use of small molecules. We therefore screened for compounds that increase BBB permeability in HTS. We modified the previously used assay (Salvador et al., 2015) for measuring paracellular permeability without the need to use transwell inserts and performed it in 96-well plates that enabled HTS. Validation of results in transwell for selected compounds confirmed the results obtained in 96-well plates. We used human CD34^+^ hematopoietic stem cells differentiated into BLECs in 96-well plates to identify compounds that affect the paracellular permeability of the BBB and therefore may potentially be used in clinical practice to increase CNS drug delivery of systemically administered drugs. From 1,278 compounds, we identified 175 substances that cause at least a 50 percent increase in BBB permeability.

Some of the here identified compounds are described in literature as permeability increasing compounds. For example, stattic, a JAK/STAT3 (JAK: Janus Kinase, STAT: Signal Transducer and Activator of Transcription 3) inhibitor that belongs to compound class “gene regulation” caused in our experiments 2-fold increase in permeability. Inhibition of STAT3 with stattic in brain microvascular endothelial cells reduced barrier integrity by lowering the TEER and increasing the permeability to FITC-Ficoll 70 (Davis et al., 2022). Rottlerin, a compound of “phosphorylation” class is an inhibitor of protein kinase C δ that caused a 2-fold increase of permeability in our experiments. Treatment with rottlerin significantly increased endothelial permeability in pulmonary microvascular endothelial cells and caused pulmonary edema *in vivo* (Klinger et al., 2007). Also in brain microvascular endothelial cells bEnd.3 rottlerin exacerbated hyperpermeability during aglycemic hypoxia (Kim et al., 2010). Sirtulins have barrier-stabilizing effects and their loss or inhibition increases the BBB permeability and exacerbates neuroinflammation (Yuan et al., 2016; Stamatovic et al., 2019). AGK2, a selective sirtulin-2 inhibitor that belongs to compound class “gene regulation” caused a 2.1 fold-increase in permeability in our experiments. Many other compounds identified in our study haven not been studied in endothelial permeability measurements yet.

Two compounds, ANI (4-amino-1,8-naphthalimide) and GW2974, which belong to the class of compounds used in the treatment of breast cancer and caused a 2.8- and 3.5-fold increase in permeability, in our experiments respectively, were selected for more detailed analysis. ANI is one of the poly (ADP-ribose) polymerases (PARPs) inhibitors. PARPs bind to DNA and catalyze the repair of single-strand DNA breaks (Muñoz-Gámez et al., 2005). PARP inhibitors bind to the active site of PARPs, preventing their dissociation from the DNA strand (Pommier et al., 2016). PARP inhibitors are used in cancer therapy as part of maintenance therapy after chemotherapy in breast carcinoma. The PARP inhibitor olaparib is currently approved as maintenance therapy in BRCA mutation carriers in adjuvant therapy for locally advanced or metastatic HER2-negative breast cancer (Tutt et al., 2021). Here, treatment of BLECs with ANI resulted in lower barrier properties in BLECs, accompanied by expression changes of the transporter and junctional protein occludin. Our results are consistent with other reports in which olaparib treatment reduced infraction volume but not cerebral edema in mice subjected to transient cerebral ischemia (Teng et al., 2016). These effects were concentration dependent. Similar effects were observed with ANI, a significant increase in permeability was concentration dependent.

GW2974, a tyrosine kinase inhibitor, is a HER-2 inhibitor and a structural analogue of clinically used tyrosine kinase inhibitor lapatinib (Sodani et al., 2012). Inhibitory effects of GW2974 on breast cancer cells have been reported (Witters et al., 2007). Furthermore, GW2974 reversed ABCG2-and ABCB1-mediated drug resistance in breast cancer cell lines (Sodani et al., 2012). Here, treatment of BLECs with GW2974 increased paracellular endothelial permeability and reduced TEER. These barrier-compromising effects were associated with altered cell-cell contact protein expression. Claudin-5 expression was reduced at the mRNA and protein levels, while expression of the other junctional proteins ZO-1 and VE-cadherin was downregulated at the protein-level only.

In conclusion, our study identified several novel and known small molecules that influence paracellular permeability of the endothelial monolayer. Further experiments to validate the effects of these small molecules should be performed *in vivo* and *in vitro*. There are some limitations to our study. In our HTS-assay, we tested only one type of brain endothelial cells. There are other *in vitro* models available that have already been successfully used to model various diseases (Helms et al., 2016; Blecharz-Lang et al., 2018; Salvador et al., 2018; Ittner et al., 2020). In addition, we used the Library of Pharmacologically Active Compounds only at a concentration of 100 µM. Therefore, selected compounds with a strong influence on BBB permeability should be tested at various concentrations on additional BBB models *in vitro* and *in vivo* to identify compounds that could be used in clinical practice. However, the clinical use of compounds that increase BBB permeability may be limited due to high side effects, adverse effects on chemotherapy treatment, and/or lack of clinical trials.

## Data Availability

The original contributions presented in the study are publicly available. This data can be found here: https://pubchem.ncbi.nlm.nih.gov/bioassay/1963498.
